# Using machine learning to improve the accuracy of genomic prediction of reproduction traits in pigs

**DOI:** 10.1186/s40104-022-00708-0

**Published:** 2022-05-17

**Authors:** Xue Wang, Shaolei Shi, Guijiang Wang, Wenxue Luo, Xia Wei, Ao Qiu, Fei Luo, Xiangdong Ding

**Affiliations:** 1 Key Laboratory of Animal Genetics and Breeding of Ministry of Agriculture and Rural Affairs, National Engineering Laboratory of Animal Breeding, College of Animal Science and Technology, China Agricultural University, Beijing, China; 2 Hebei Province Animal Husbandry and Improved Breeds Work Station, Shijiazhuang, Hebei China; 3 Zhangjiakou Dahao Heshan New Agricultural Development Co., Ltd, Zhangjiakou, Hebei China

**Keywords:** Genomic prediction, Machine learning, Pig, Prediction accuracy

## Abstract

**Background:**

Recently, machine learning (ML) has become attractive in genomic prediction, but its superiority in genomic prediction over conventional (ss) GBLUP methods and the choice of optimal ML methods need to be investigated.

**Results:**

In this study, 2566 Chinese Yorkshire pigs with reproduction trait records were genotyped with the GenoBaits Porcine SNP 50 K and PorcineSNP50 panels. Four ML methods, including support vector regression (SVR), kernel ridge regression (KRR), random forest (RF) and Adaboost.R2 were implemented. Through 20 replicates of fivefold cross-validation (CV) and one prediction for younger individuals, the utility of ML methods in genomic prediction was explored. In CV, compared with genomic BLUP (GBLUP), single-step GBLUP (ssGBLUP) and the Bayesian method BayesHE, ML methods significantly outperformed these conventional methods. ML methods improved the genomic prediction accuracy of GBLUP, ssGBLUP, and BayesHE by 19.3%, 15.0% and 20.8%, respectively. In addition, ML methods yielded smaller mean squared error (MSE) and mean absolute error (MAE) in all scenarios. ssGBLUP yielded an improvement of 3.8% on average in accuracy compared to that of GBLUP, and the accuracy of BayesHE was close to that of GBLUP. In genomic prediction of younger individuals, RF and Adaboost.R2_KRR performed better than GBLUP and BayesHE, while ssGBLUP performed comparably with RF, and ssGBLUP yielded slightly higher accuracy and lower MSE than Adaboost.R2_KRR in the prediction of total number of piglets born, while for number of piglets born alive, Adaboost.R2_KRR performed significantly better than ssGBLUP. Among ML methods, Adaboost.R2_KRR consistently performed well in our study. Our findings also demonstrated that optimal hyperparameters are useful for ML methods. After tuning hyperparameters in CV and in predicting genomic outcomes of younger individuals, the average improvement was 14.3% and 21.8% over those using default hyperparameters, respectively.

**Conclusion:**

Our findings demonstrated that ML methods had better overall prediction performance than conventional genomic selection methods, and could be new options for genomic prediction. Among ML methods, Adaboost.R2_KRR consistently performed well in our study, and tuning hyperparameters is necessary for ML methods. The optimal hyperparameters depend on the character of traits, datasets etc.

**Supplementary Information:**

The online version contains supplementary material available at 10.1186/s40104-022-00708-0.

## Background

Genomic selection (GS) has been widely recognized and successfully implemented in animal and plant breeding programs [1–3]. It has been reported that the breeding costs of dairy cattle using GS were 92% lower than those of traditional progeny testing [4]. At present, the genetic gain rate of the annual yield traits of US Holstein dairy cattle has increased from approximately 50% to 100% [5]. The accuracy of GS is impacted by a number of factors, such as analytical methods of genomic prediction, reference population size, marker density, and heritability values. Currently, parametric methods are most commonly used for livestock and poultry genomic selection, mainly including genomic BLUP (GBLUP) [6], single-step GBLUP (ssGBLUP) [7, 8], ridge regression (RR) [9], least absolute shrinkage and selection operator (LASSO) [10], and Bayesian regression models [11, 12] with the difference mainly depending on the prior distribution of marker effects. Nevertheless, these linear models usually only take into account the additive inheritance and ignore the complex nonlinear relationships that may exist between markers and phenotypes (e.g. epistasis, dominance, or genotype-by-environment interactions). In addition, parametric methods usually provide limited flexibility for handling nonlinear effects in high-dimensional genomic data, resulting in large computational demands [13]. However, studies have shown that considering nonlinearity may enhance the genomic prediction ability of complex traits [14]. Therefore, new strategies should be explored to more accurately estimate genomic breeding values.

Driven by applications in intelligent robots, self-driving cars, automatic translation, face recognition, artificial intelligence games and medical services, machine learning (ML) has gained considerable attention in the past decade. Some characteristics of ML methods make them potentially attractive for dealing with high-order nonlinear relationships in high-dimensional genomic data, e.g. allowing the number of variables larger than the sample size [15], capable of capturing the hidden relationship between genotype and phenotype in an adaptive manner, and imposing little or no specific distribution assumptions about the predictor variables as GBLUP and Bayesian methods [16, 17].

Studies have shown that random forest (RF), support vector regression (SVR), kernel ridge regression (KRR) and other machine learning methods have advantages over GBLUP and Bayes B [18–20]. Ornella et al. compared the genomic prediction performance of support vector regression, random forest regression, reproducing kernel Hilbert space (RKHS), ridge regression, and Bayesian Lasso in maize and wheat datasets with different trait-environment combinations, and found that RKHS and random forest regression were the best [21]. González-Camacho et al. reported that the support vector machine (SVM) with linear kernel performed the best in comparison with other ML methods and linear models in the genomic prediction of the rust resistance of wheat [20]. Additionally, ML methods have also been widely used in the fields of gene screening, genotype imputation, and protein structure and function prediction [22–25], demonstrating its superiority as well. However, one challenge for ML is choosing the optimum ML method as a series of ML methods have been proposed and each has its own characteristics and shows different prediction abilities in different datasets and traits.

Therefore, the objectives of this study were to 1) assess the performance of ML methods in genomic prediction in comparison with existing prevail methods of GBLUP, ssGBLUP, and BayesHE and 2) evaluate the efficiency of different ML methods to explore the ideal ML method for genomic prediction.

## Materials and methods

### Ethics statement

The whole procedure for blood sample collection was carried out in strict accordance with the protocol approved by the Animal Care and Use Committee of China Agricultural University (Permit Number: DK996).

### Population and phenotypes

A purebred Yorkshire pig population from DHHS, a breeding farm in Hebei Province, China, was studied. Animals from this farm were descendants of Canadian Yorkshires, and they were reared under the same feeding conditions. A total of 2566 animals born between 2016 and 2020 were sampled, their 4274 reproductive records of the total number of piglets born (TNB) and the number of piglets born alive (NBA) with delivery dates ranging from 2017 to 2021 were available, and 3893 animals were traced back to construct the pedigree relationship matrix (A matrix). The numbers of full-sib and half-sib families were 339 and 301, respectively. A single-trait repeatability model was used to estimate the heritability. The fixed effect included herd-year-season, and random effects included additive genetic effects, random residuals, and permanent environment effects of sows (environmental effects affecting litter size across parities of sows). The information of animals, phenotypes and genetic components, as well as the estimated heritability, are listed in Table 1. The estimated heritability of TNB and NBA were both 0.12.

**Table 1:** Summary of two reproduction traits of Yorkshire pigs

Trait^a^	Number of records	Birth year	Genotyped animals	Mean	SD	Minimum	Maximum	σ^2^_a_	σ^2^_e_	h^2^(SE)
TNB	4274	2016–2020	2566	13	3.38	3	24	1.26	8.95	0.12 (0.034)
NBA	4274	2016–2020	2566	12	3.13	3	24	0.98	7.13	0.12 (0.032)

^a^ TNB: total number of piglets born; NBA: number of piglets born alive

*SE* standard error

### Derivation of corrected phenotypes

To avoid double counting of parental information, the corrected phenotypes (y_c_) derived from the estimated breeding values (EBVs) were used as response variables in genomic prediction. The pedigree-based BLUP and single-trait repeatability model was performed to estimate the breeding values for each trait separately.

1 $$ y= Xb+{Z}_aa+{Z}_{pe} pe+e, $$

where *y* was the vector of raw phenotypic values; *b* was the vector of fixed effects including herd-year-season, in which season consisted of four levels (1st = December to February; 2nd = March to May; 3rd = June to August; 4th = September to November); *a* was the vector of additive genetic effects; *pe* was the vector of permanent environment effects of sows; and *e* was the vector of random error. *X*, *Z*_*a*_, and *Z*_*pe*_ are the incidence matrices linking *b*, *a* and *pe* to *y*. The random effects were assumed to be normally distributed as follows: *a* ~ N (0, Aσ^2^_a_), *pe* ~ N (0, Iσ^2^_pe_), and e ~ N (0, Iσ^2^_e_), where A was the pedigree-based relationship matrix; I was the identity matrix; and σ^2^_a_, σ^2^_pe_, and σ^2^_e_ were the variances of additive genetic effects, permanent environment effects of sows, and residuals, respectively. A total of 3893 individuals were traced to construct matrix A. Their EBVs were calculated using the DMUAI procedure of the DMU software [26]. The *y*_*c*_ were calculated as EBV plus the average estimated residuals for multiple parties of a sow following Guo et al. [27].

### Genotype data and imputation

Two kinds of 50 K define SNP panels, PorcineSNP50 BeadChip (Illumina, CA, USA) and GenoBaits Porcine SNP 50 K (Molbreeding, China) were used for genotyping. A total of 1189 sows were genotyped with the PorcineSNP50 BeadChip, which included 50,697 SNPs across the genome, and 1978 individuals were genotyped using the GenoBaits Porcine SNP 50 K with 52,000 SNPs. There were 30,998 common SNPs between these two SNP panels, and 601 individuals were genotyped with both SNP panels; therefore, 2566 genotyped individuals were finally used for further analysis, including 1189 animals with the PorcineSNP50 BeadChip and 1377 pigs with the GenoBaits Porcine SNP 50 K. The animals genotyped with GenoBaits Porcine SNP 50 K were imputed to the PorcineSNP50 BeadChip using Beagle 5.0 [28]. The reference population size for genotype imputation was 3720. Imputation accuracy was assessed by the dosage R-squared measure (DR2), which is the estimated squared correlation between the estimated allele dose and the true allele dose. The genotype correlation (COR) and the genotype concordance rate (CR) were also calculated based on the 601 overlapped animals to evaluate the imputation accuracy. After imputation, quality control of the genotype was carried out using PLINK software [29]. SNPs with a minor allele frequency (MAF) lower than 0.01 and call rate lower than 0.90 were removed, and individuals with call rates lower than 0.90 were excluded. Finally, all animals and 44,922 SNPs on autosomes remained for further analysis.

### Statistical models

GBLUP, ssGBLUP, Bayesian Horseshoe (BayesHE) and four ML regression methods, support vector regression (SVR), kernel ridge regression (KRR), random forest (RF), and Adaboost.R2 were used to perform genomic prediction.

### GBLUP

$$ {\boldsymbol{y}}_{\boldsymbol{c}}=\mathbf{1}\boldsymbol{\mu } +\boldsymbol{Zg}+\boldsymbol{e} $$

in which ***y***_***c***_ is the vector of corrected phenotypes of genotyped individuals. ***μ*** is the overall mean, **1** is a vector of 1 s, ***g*** is the vector of genomic breeding values, ***e*** is the vector of random errors, and **Z** is an incidence matrix allocating records to ***g***. The distributions of random effects were: ***g*** ~ N (**0**, **G** ${\sigma}_g^2$) and ***e*** ~ N (**0**, **I** ${\sigma}_e^2$), where **G** was the genomic relationship matrix (G matrix), and ${\sigma}_g^2$ and ${\sigma}_e^2$ were the additive genetic variance and the residual variance, respectively.

### ssGBLUP

ssGBLUP had the same expression as GBLUP, except that it used ***y***_***c***_ of both genotyped and nongenotyped individuals by combining the G matrix and A matrix. It was assumed that ***g*** followed a normal distribution N (**0**, **H** ${\sigma}_g^2$). The inverse of matrix H was:

$$ {H}^{-1}=\left[\begin{array}{cc}{G}_w^{-1}-{A}_{22}^{-1}& 0\\ {}0& 0\end{array}\right]+{A}^{-1} $$

To prevent the problem that the singular matrix cannot be inverted, *G*_*w*_ *= (1-w) G*_*a*_ *+ wA*_*22*_, and *w* was equal to 0.05 [30].

### BayesHE

BayesHE was developed by Shi. et al. [31], it was based on global-local priors to increase the flexibility and adaptability of the Bayesian model. In this study, the first form of BayesHE (BayesHE1) was used [31], and the Markov chain Monte Carlo (MCMC) chain was run for 50,000 cycles, with the first 20,000 cycles being discarded as burn-in and every 50 samples of the remaining 30,000 iterations saved to infer posterior statistics. In-house scripts written in Fortran 95 were used for BayesHE analyses [31], and the DMUAI procedure implemented in DMU software [26] was used for GBLUP and ssGBLUP analyses.

### Support vector regression

Support vector machine (SVM) was based on statistical learning theory. SVR was the application of SVM in regression for dealing with quantitative responses, which used a linear or nonlinear kernel function to map the input space (the marker dataset) to a higher dimensional feature space [32], and performed modelling and prediction on the feature space. In other words, we can build a linear model in the feature space to deal with regression problems. The model formulation of SVR can be expressed as:

2 $$ f(x)={\beta}_0+h{(x)}^T\beta $$

in which *h*(*x*)^*T*^*β* is the kernel function, *β* is the vector of weights, and *β*_0_ is the bias. Generally, the formalized SVR was given by minimizing the following restricted loss function:

3 $$ \underset{\beta_0,\beta }{\mathit{\min}}\frac{1}{2}{\left\Vert \beta \right\Vert}^2+C{\sum}_{i=1}^nV\left({y}_i-f\left({x}_i\right)\right), $$

in which

4 $$ {V}_{\varepsilon }(r)=\left\{\begin{array}{c}0, if\left|r\right|<\varepsilon \\ {}\left|r\right|-\varepsilon, otherwise\end{array}\right.. $$

*V*_*ε*_(*r*) is the *ε*-insensitive loss and *C* (“cost parameter”) is the regularization constant that controls the trade-off between prediction error and model complexity. *y* is a quantitative response, and ||·|| is the norm in Hilbert space. After optimization, the final form of SVR can be written as:

5 $$ f(x)={\sum}_{i=1}^m\left({\hat{a}}_i-{a}_i\right)k\left(x,{x}_i\right), $$

in which *k*(*x*_*i*_, *x*_*j*_) = *ϕ*(*x*_*i*_)^*T*^*ϕ*(*x*_*j*_) is the kernel function. In this research, grid search was used to find the best kernel function and the optimal hyperparameters of *C* and gamma. An internal fivefold cross-validation (5-fold CV) strategy was performed to tune the hyperparameters when performing a grid search.

### Kernel ridge regression

Kernel ridge regression (KRR) is a nonlinear regression method that can effectively discover the nonlinear structure of the data [33]. KRR uses a nonlinear kernel function to map the data to a higher dimensional kernel space, and then builds a ridge regression model to make the data linearly separable in this kernel space. The linear function in the kernel space was selected according to the mean squared error loss of ridge regularization [33]. The final KRR prediction model can be written as:

6 $$ y\left({x}_i\right)={k}^{\prime }{\left(K+\lambda I\right)}^{-1}\hat{y} $$

where *λ* is the regularization constant, and *K* is the Gram matrix with entries *K*_*ij*_ = *K*(*x*_*i*_, *x*_*j*_) = *ϕ*(*x*_*i*_) · *ϕ*(*x*_*j*_)^*T*^; thus, for n training samples, the obtained kernel matrix is:

7 $$ K={\left[\begin{array}{cccc}K\left({x}_1,{x}_1\right)& K\left({x}_1,{x}_2\right)& \cdots & K\left({x}_1,{x}_n\right)\\ {}K\left({x}_2,{x}_1\right)& K\left({x}_2,{x}_2\right)& \cdots & K\left({x}_2,{x}_n\right)\\ {}\vdots & \vdots & \vdots & \vdots \\ {}K\left({x}_n,{x}_1\right)& K\left({x}_n,{x}_2\right)& \cdots & K\left({x}_n,{x}_n\right)\end{array}\right]}_{n\ast n} $$

*I* is the identity matrix, *k*^′^ = *K*(*x*_*i*_, *x*_*j*_) with *j = 1,2,3, …,n, n* is the number of training samples, and *x*_*i*_ is the test sample. In the expanded form,

8 $$ k=\left[\begin{array}{c}K\left({x}_i,{x}_1\right)\\ {}K\left({x}_i,{x}_2\right)\\ {}\vdots \\ {}K\left({x}_i,{x}_n\right)\end{array}\right] $$

The grid search was used to find the most suitable kernel function and *λ* in this study, and an internal 5-fold CV strategy was used for tuning the hyperparameters.

### Random forest

Random forest (RF) is an ML method that uses voting or the average of multiple decision trees to determine the classification or predicted values of new instances [34]. Random forest was essentially a collection of decision trees, and each decision tree was slightly different from other trees. Random forest reduced the risk of overfitting by averaging the prediction results of many decision trees [20]. Random forest regression can be written in the following form:

9 $$ y=\frac{1}{M}{\sum}_{m=1}^M{t}_m\left({\psi}_m\left(y:X\right)\right) $$

in which *y* is the predicted value of random forest regression, *t*_*m*_(*ψ*_*m*_(*y* : *X*)) is an individual regression tree, and *M* is the number of decision trees in the forest. The prediction was obtained by passing down the predictor variables in the flowchart of each tree, and the corresponding estimated value at the terminal node was used as the predicted value. Finally, the predictions of each tree in RF were averaged to calculate the final prediction of unobserved data. The grid search was used to find the most suitable hyperparameter *M* and the maximum depth of the tree, and the inner 5-fold CV was performed to tune the hyperparameters.

### Adaboost.R2

Adaboost.R2 is an ad hoc modification of Adaboost. R and an extension of Adaboost.M2 created to deal with regression problems, which repeatedly used a regression tree as a weak learner followed by increasing the weights of incorrectly predicted samples and decreasing the weights of correctly predicted samples. It builds a “committee” by integrating multiple weak learners [35], making its prediction effect better than those of weak learners. Adaboost.R2 regression model can be written as:

10 $$ y=\mathit{\operatorname{inf}}\left[y\in Y:{\sum}_{t:{f}_t(x)\le y}\mathit{\log}\frac{1}{\varepsilon_t}\ge \frac{1}{2}{\sum}_t\mathit{\log}\frac{1}{\varepsilon_t}\right], $$

where *y* is the predicted value, *f*_*t*_(*x*) is the predicted value of the t-th weak learner, *ε*_*t*_ is the error rate of *f*_*t*_(*x*) and ${\upvarepsilon}_t={\overline{L}}_t\div \left(1-{\overline{L}}_t\right)$, ${\overline{L}}_t$ is the average loss and ${\overline{L}}_t={\sum}_{i=1}^m{L}_t(i){D}_t(i),$ *L*_*t*_(*i*) is the error between the actual observation value and the predicted value of the i-th predicted individual, and *D*_*t*_(*i*) is the weight distribution of *f*_*t*_(*x*). After *f*_*t*_(*x*) is trained, the weight distribution *D*_*t*_(*i*) becomes *D*_*t* + 1_(*i*),

11 $$ {D}_{t+1}(i)=\frac{D_t(i){\beta}_t^{\left(1-{L}_t(i)\right)}}{Z_t}, $$

in which *Z*_*t*_ is a normalization factor chosen such that *D*_*t* + 1_(*i*) will be a distribution. In the current study, SVR and KRR were used as weak learners of Adaboost.R2.

For these four ML methods, the vectors of genotypes (coded as 0, 1, 2) were the input independent variables, corrected phenotypes *y*_*c*_ was used as the response variable, and the Sklearn package for Python (V0.22) was used for genomic prediction. We sought the optimal hyperparameter combination from a grid of values with different hyperparameter combinations, and the combination in the grid with the highest Pearson correlation was selected as the optimal hyper-parameter in each fold (grid search). Meanwhile, the optimal hyperparameters for SVR, KRR, RF and Adaboost.R2 in CV according to the grid search are shown in Table 2.

**Table 2:** The optimal hyperparameters of each ML model obtained through a grid search for TNB and NBA traits in 20 replicates of 5-fold CV

Method	Optimal hyperparameters^a^
SVR	kernel = ‘rbf’, C = 7, gamma = 0.0001
KRR	kernel = ‘rbf’, *λ* =0.1, gamma = 0.0001
RF	n_estimators = 250, max_depth = None
Adaboost.R2_SVR	n_estimators = 50, kernel = ‘rbf’, C = 7, gamma = 0.0001
Adaboost.R2_KRR	n_estimators = 50, kernel = ‘rbf’, *λ* =0.01, gamma = 0.0001

^a^ Optimal hyperparameters: The optimal hyperparameters of each machine learning method obtained by using a grid search

### Accuracy of genomic prediction

Fivefold cross-validation (5-fold CV) was used to estimate the accuracies of genomic prediction, in which 2566 individuals were randomly split into five groups with 513 individuals each. For each CV, four of the five groups were defined as the reference population, and the remaining group was treated as the validation population. The genotyped reference and validation sets in each replicate of 5-fold CV were the same for all methods, and it should be noted that nongenotyped individuals were added to the reference population in ssGBLUP. For all methods, the accuracy of genomic prediction was calculated as the Pearson correlation of *y*_*c*_ (corrected phenotypes) and PV (predicted values). In addition, the prediction unbiasedness was also calculated as the regression of *y*_*c*_ on PV of the validation population. The 5-fold CV scheme was repeated 20 times, and the overall prediction accuracy and unbiasedness were the averages of 20 replicates. The Hotelling-Williams Test [36] was performed to compare the prediction accuracy of different methods after parameter optimization.

Meanwhile, prediction ability metrics, e.g.*,* mean squared error (MSE) and mean absolute error (MAE), were also used to evaluate the performance of regression models in the present study. MSE can take both prediction accuracy and bias into account [37], and the smaller the value of MSE is, the better the accuracy of the model to describe the experimental data. The MAE could better reflect the actual situation of the predicted value error. Their formulas can be written as follows.

12 $$ MSE=\frac{1}{m}{\sum \limits}_{i=1}^m{\left({f}_i-{y}_i\right)}^2,\kern0.5em and\kern0.5em MAE=\frac{1}{m}{\sum \limits}_{i=1}^m\left|{f}_i-{y}_i\right| $$

where *m* represents the number of animals in each CV test fold of 5-fold CV, *f* is the vector of predicted values (PV) and *y* is the vector of observed values (y_c_). The final MSE and MAE were the average of 20 replicates.

In addition, to be more in line with the actual situation of genomic selection, we compared ML methods and traditional genomic selection methods in using early-generation animals to predict the performance of animals of later generations. Therefore, the younger animals born after January 2020 were chosen as the validation population, and the population sizes of the reference and validation were 2222 and 344, respectively. The accuracy of genomic prediction was evaluated as *r (y*_*c*_*,* PV*)*, the Pearson correlation between corrected phenotypes *y*_*c*_ and predicted values PV.

## Results

### Genotype imputation accuracy

**Fig. 1:**
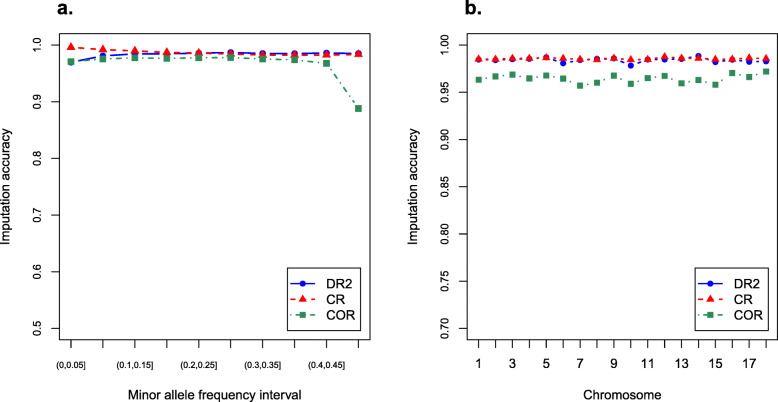
Imputation accuracy. Imputation accuracy of GenoBaits Porcine SNP 50 K to PorcineSNP50 BeadChip at different minor allele frequency (MAF) intervals (**a**) and chromosomes (**b**). DR2, the estimated squared correlation between the estimated allele dose and the true allele dose; Genotype concordance rate (CR), the ratio of correctly imputed genotypes; Genotype correlation (COR), the correlation coefficient between the imputed variants and the true variants

Figure 1 illustrates the accuracy of imputing GenoBaits Porcine SNP 50 K to PorcineSNP50 BeadChip across minor allele frequency (MAF) intervals and chromosomes. DR2, CR and COR were not sensitive to MAF except that COR was lower when the MAF was less than 0.05 and in the range of 0.45 to 0.5 (Fig. 1a). DR2, CR and COR on each chromosome were 0.978 ~ 0.988, 0.984 ~ 0.988 and 0.957 ~ 0.972, respectively, and no significant differences were observed in DR2, CR and COR between chromosomes (Fig. 1b). In the same scenarios, the COR values were smaller than those of DR2 and CR. The averaged DR2, CR and COR across all variants were 0.984, 0.985 and 0.964, respectively, indicating that the imputation was sufficiently accurate to analyse the two SNP panels together.

### Accuracy of genomic prediction in cross-validation

#### Comparison of ML methods with (ss) GBLUP and BayesHE

Table 3 shows the prediction accuracies and unbiasedness of the ML methods, (ss) GBLUP and BayesHE on traits of TNB and NBA in 20 replicates of 5-fold CV. The accuracies of the ML methods after tuning the hyperparameters were significantly (*P* < 0.05) higher than those of (ss) GBLUP and BayesHE. The improvements of ML methods over GBLUP, ssGBLUP and BayesHE were 19.3%, 15.0% and 20.8% on average, ranging from 8.9% to 24.0%, 7.6% to 17.5% and 11.1% to 24.6%, respectively. For trait TNB, compared with that of GBLUP, the average accuracy of all ML methods was improved, and support vector regression (SVR) showed an improvement of 19.0%, similar to the outcomes of kernel ridge regression (KRR) and Adaboost.R2 based on SVR and KRR, which obtained improvements of 18.1% and 17.7%, respectively; random forest (RF) yielded the lowest improvement of 8.9%. The similar advantages of ML were also over ssGBLUP and the improvements of SVR, KRR, RF, Adaboost.R2_SVR and Adaboost.R2_KRR were 17.5%, 17.5%, 7.6%, 16.7% and 16.3%, respectively. ML methods gained the largest advantage over BayesHE, the accuracy from SVR, KRR, RF, Adaboost.R2_SVR and Adaboost.R2_KRR were improved by 21.4%, 21.4%, 11.1%, 20.6% and 20.2%, respectively, compared with BayesHE. For trait NBA, although ML methods still performed better than GBLUP, ssGBLUP and BayesHE, Adaboost.R2_KRR gained the largest improvement in all comparisons, and KRR obtained the second largest improvement. SVR and Adaboost.R2 based on SVR yielded the same improvements on GBLUP, ssGBLUP and BayesHE. RF still gained the lowest improvement compared with other ML methods.

**Table 3:** Accuracies and unbiasedness of genomic prediction on TNB and NBA from seven methods in 20 replicates of 5-fold CV

Hyper-parameters	Method	TNB^1^		NBA^2^	
		Accuracy^3^	Unbiasedness^4^	Accuracy^3^	Unbiasedness^4^
	GBLUP	0.248^a^ ± 0.026	0.958 ± 0.132	0.208^a^ ± 0.025	0.931 ± 0.142
	ssGBLUP	0.251^a^ ± 0.026	0.901 ± 0.121	0.221^ab^ ± 0.026	0.844 ± 0.113
	BayesHE	0.243^a^ ± 0.025	1.015 ± 0.148	0.207^a^ ± 0.026	1.009 ± 0.171
Tuning	SVR	0.295^b^ ± 0.025	1.23 ± 0.119	0.254^b^ ± 0.023	1.106 ± 0.11
	KRR	0.295^b^ ± 0.025	1.266 ± 0.125	0.256^b^ ± 0.023	1.151 ± 0.113
	RF	0.270^ab^ ± 0.029	1.229 ± 0.152	0.248^ab^ ± 0.028	1.188 ± 0.147
	Adaboost.R2_SVR	0.293^b^ ± 0.025	1.363 ± 0.138	0.254^b^ ± 0.024	1.256 ± 0.131
	Adaboost.R2_KRR	0.292^b^ ± 0.025	1.344 ± 0.136	0.258^b^ ± 0.024	1.249 ± 0.129
Default	SVR	0.255 ± 0.027	1.275 ± 0.147	0.224 ± 0.023	1.098 ± 0.126
	KRR	0.264 ± 0.025	1.007 ± 0.108	0.222 ± 0.024	0.879 ± 0.101
	RF	0.246 ± 0.028	1.064 ± 0.142	0.225 ± 0.027	1.002 ± 0.128
	Adaboost.R2_SVR	0.273 ± 0.024	0.998 ± 0.106	0.228 ± 0.026	0.822 ± 0.099
	Adaboost.R2_KRR	0.254 ± 0.024	0.759 ± 0.085	0.209 ± 0.027	0.636 ± 0.085

^1^ TNB: total number of piglets born

^2^ NBA: number of piglets born alive

^3^ Accuracy: the correlation between corrected phenotypes and predicted values of the validation population;

^4^ Unbiasedness: the regression of corrected phenotypes onto the predicted values

The different superscript of accuracy indicates the significant difference by the Hotelling-Williams test

Meanwhile, GBLUP, ssGBLUP and BayesHE had similar performance, and no significant differences in prediction accuracy were found among them. Nevertheless, ssGBLUP produced an average improvement of 3.7% compared with GBLUP (1.2% for TNB; 6.3% for NBA), while less bias was observed by GBLUP in all scenarios. BayesHE yielded similar accuracy to GBLUP (0.243 and 0.248 for TNB; 0.207 and 0.208 for NBA), but the unbiasedness of BayesHE was much closer to 1 (1.015 for TNB; 1.009 for NBA).

On the other hand, the mean squared error (MSE) and mean absolute error (MAE) were also used to assess the performance of different methods. As shown in Table 4, after tuning the hyperparameters, the ML methods were generally superior to GBLUP, ssGBLUP and BayesHE in terms of MSE and MAE. For two reproduction traits TNB and NBA, all ML methods yielded lower MSE and MAE than GBLUP, ssGBLUP and BayesHE. The performance of GBLUP, ssGBLUP and BayesHE was very close, and ssGBLUP produced a slightly lower MSE (5.26 for TNB; 3.95 for NBA) and MAE (1.748 for TNB; 1.532 for NBA) among these three methods, while they were still higher than those obtained from RF, which performed the worst among the four ML methods and generated 5.212 and 3.901 of MSE and 1.747 and 1.527 of MAE on TNB and NBA, respectively.

**Table 4:** Mean squared error (MSE) and mean absolute error (MAE) of seven methods for TNB and NBA as assessed with 20 replicates of 5-fold CV

Hyperparameters	Method	TNB		NBA	
		MSE	MAE	MSE	MAE
	GBLUP	5.259	1.749	4.168	1.606
	ssGBLUP	5.26	1.748	3.95	1.532
	BayesHE	5.32	1.763	4.023	1.556
Tuning	SVR	5.129	1.730	3.880	1.521
	KRR	5.134	1.731	3.876	1.521
	RF	5.212	1.747	3.901	1.527
	Adaboost.R2_SVR	5.158	1.739	3.892	1.528
	Adaboost.R2_KRR	5.153	1.737	3.883	1.526
Default	SVR	5.271	1.748	3.956	1.522
	KRR	5.21	1.743	3.944	1.531
	RF	5.266	1.756	3.93	1.531
	Adaboost.R2_SVR	5.202	1.75	3.95	1.541
	Adaboost.R2_KRR	5.309	1.771	4.04	1.566

#### Comparison among ML methods

Tables 3 and 4 indicate that the ML methods performed better than GBLUP, ssGBLUP and BayesHE. They also showed that RF had the lowest accuracy even though no significant differences were observed among the ML methods in this study. After tuning the parameters, the accuracies of SVR, KRR, Adaboost.R2_SVR and Adaboost.R2_KRR was improved by an average of 5.8%, 6.2%, 5.5% and 6.1% compared to RF, ranging from 8.1% to 9.3% for TNB and from 2.4% to 4.0% for NBA. For TNB, SVR and KRR showed the highest accuracies (0.295 for both), and Adaboost.R2_KRR yielded the highest accuracies on NBA (0.258). In the comparison of unbiasedness, SVR produced the lowest genomic prediction bias, and the regression coefficient was close to 1.0, while Adaboost.R2 method with both base learner SVR and KRR produced larger bias. As a trade-off between accuracy and unbiasedness, SVR and KRR had the most robust prediction ability, which was also confirmed by the results of MSE and MAE, in which SVR and KRR had the smallest MSE and MAE in all scenarios.

It should be noted that the better performance of the ML methods was acquired by tuning the hyperparameters (Tables 2,3). Compared with using hyperparameters set to default, the accuracy was improved by 14.3% on average from the ML methods with optimal hyperparameters; the accuracy of SVR, KRR, RF and Adaboost.R2 with optimal hyperparameters improved the genomic prediction accuracies for TNB by 15.7%, 11.7%, 9.8% and 15.0%, respectively; and for NBA, the improvements were 13.4%, 15.3%, 10.2% and 23.4%, respectively. For unbiasedness, except for SVR on TNB, the unbiasedness of all ML methods using the default parameters was lower than the unbiasedness using the optimal parameters. On the other hand, Tables 3 and 4 indicate that ML methods with default hyperparameters did not yield advantages over GBLUP, ssGBLUP and BayesHE.

### Accuracy of genomic prediction in predicting younger animals

Table 5 presents the accuracy and MSE of genomic prediction on TNB and NBA by applying different methods to predict younger animals. On the one hand, a similar trend was obtained for GBLUP, BayesHE and ssGBLUP as in CV. GBLUP performed comparably with BayesHE, while ssGBLUP yielded higher accuracies and lower MSE than GBLUP and BayesHE for both traits. On the other hand, different from the results in CV, the superiority of ML methods with optimal hyperparameters was not significant in predicting younger animals, although they still improved the accuracies and reduced the MSE compared with the outcomes when using the default hyperparameters. Table 5 indicates that Adaboost.R2_KRR and RF still outperformed GBLUP and BayesHE as was demonstrated in the CV, ssGBLUP performed comparably with RF, and ssGBLUP yielded slightly higher accuracy and lower MSE than Adaboost.R2_KRR in the prediction of TNB; in contrast, for NBA, Adaboost.R2_KRR performed significantly better than ssGBLUP. Meanwhile, after tuning the parameters, RF and KRR obtained higher accuracies and lower MSE than GBLUP and BayesHE, respectively. The performance of RF was significantly improved, and it performed better than that of KRR and SVR. In the prediction of younger animals, SVR with either default hyperparameters or optimal hyperparameters performed the worst, which was different from its performance in the CV.

**Table 5:** Accuracy and mean squared error (MSE) of genomic prediction of TNB and NBA in younger individuals from seven methods

Hyperparameters	Method	TNB^1^			NBA^2^		
		Accuracy^3^	MSE	Optimal hyperparameters^4^	Accuracy^3^	MSE	Optimal hyperparameters^4^
	GBLUP	0.355^ab^	11.598	–	0.264^ab^	10.203	–
	ssGBLUP	0.408^b^	11.221	–	0.288^ab^	9.974	–
	BayesHE	0.357^ab^	11.566	–	0.262^ab^	10.143	–
Tuning	SVR	0.307^a^	11.488	kernel = ‘rbf’; gamma = 0.00005; C = 14	0.229^a^	10.235	kernel = ‘rbf’; gamma = 0.00005; C = 13
	KRR	0.362^ab^	11.367	kernel = ‘rbf’; gamma = 0.000001; λ = 0.07	0.266^ab^	10.121	kernel = ‘rbf’; gamma = 0.000001; λ = 0.12
	RF	0.385^ab^	11.337	n_estimators = 430; max_depth = None	0.285^ab^	10.116	n_estimators = 400; max_depth = None
	Adaboost.R2_KRR	0.395^b^	11.254	n_estimators = 70; kernel = ‘rbf’, gamma = 0.00001, λ = 1	0.328^b^	9.794	n_estimators = 60; kernel = ‘rbf’, gamma = 0.00001, λ = 0.9
Default	SVR	0.271	11.858	–	0.17	10.37	–
	KRR	0.346	11.538	–	0.259	10.158	–
	RF	0.26	11.867	–	0.179	10.335	–
	Adaboost.R2_KRR	0.36	11.392	–	0.322	9.797	–

^1^ TNB: total number of piglets born

^2^ NBA: number of piglets born alive

^3^ Accuracy: the correlation between corrected phenotypes and predicted values of the validation population;

^4^Optimal hyperparameters: The optimal hyper-parameters of each machine learning method obtained by using grid search

The different superscript of accuracy indicates the significant difference by the Hotelling-Williams test

### Computing time

The average computation time to complete each fold in CV for each genomic prediction method is demonstrated in Table 6. Running time of the methods was measured in minutes on an HP server (CentOS Linux 7.9.2009, 2.5 GHz Intel Xeon processor and 515G total memory). Among all methods, KRR was the fastest algorithm; it took an average of 1.16 min in each fold of CV to complete the analysis, requiring considerably less time than GBLUP (2.07 min) and ssGBLUP (3.23 min). The computing efficiency of SVR (5.28 min) and Adaboost.R2_KRR (5.16 min) was comparable to that of KRR, GBLUP and ssGBLUP. However, RF (53.45 min) and Adaboost.R2_SVR (85.34 min) ran slowly among the ML methods. Adaboost.R2 based on KRR (Adaboost.R2_KRR) was much more time-saving than Adaboost.R2_SVR. Since the MCMC algorithm required more iteration time to reach convergence, BayesHE was the slowest as expected, and it took 226.12 min for each fold of CV.

**Table 6:** Average computing time to complete each fold of 5-fold CV according to different genomic prediction methods

Method	TNB	NBA
GBLUP	2 min 6 s	2 min 2 s
ssGBLUP	3 min 12 s	3 min 16 s
BayesHE	3 h 57 min 1 s	3 h 35 min 13 s
SVR	5 min 27 s	5 min 7 s
KRR	1 min 4 s	1 min 16 s
RF	50 min 38 s	56 min 16 s
Adaboost.R2_SVR	1 h 35 min 13 s	1 h 15 min 28 s
Adaboost.R2_KRR	5 min 3 s	5 min 16 s

## Discussion

Our results elucidated that ssGBLUP performed better than GBLUP in terms of accuracy in all scenarios investigated, which was consistent with previous studies [27, 38–40]. This could be explained by the fact that GBLUP utilized phenotypic information only from genotyped individuals, while ssGBLUP simultaneously used information from both genotyped and nongenotyped individuals to construct a genotype-pedigree relationship matrix (H matrix). Since nongenotyped individuals were related to individuals in the validation population on the pedigree, ssGBLUP took advantage of the phenotypic information of the whole population to obtain better prediction. However, in our research utilizing 5-fold CV and predicting younger animals, ssGBLUP produced only slightly higher accuracies for the two reproduction traits. The lower improvement of ssGBLUP may be due to the following reasons. (I) Weak relationship between the nongenotyped reference population and genotyped candidates in the pedigree. In our study, only 143 of the 789 nongenotyped reference population used by ssGBLUP had pedigree information, and only 46 and 40 individual sires and dams were included in the 2566 genotyped individuals, indicating that the relationship between nongenotyped reference animals and genotyped candidates was weak, making a small contribution to the genomic prediction. Li et al. [39] showed that the improvement of ssGBLUP over GBLUP on accuracy was almost entirely contributed by nongenotyped close relatives of candidates. It can also be observed in Additional file 1: Fig. S1 that the greater the weight of the A matrix, the lower the accuracy, indicating that the information obtained from pedigree is limited, resulting in ssGBLUP not exerting its advantages greatly. (II) The low heritability of TNB and NBA. In this study, the heritability for the two traits were both 0.12, which was generally consistent with other reports [27, 41, 42]; therefore, sufficient accuracy could not be achieved with the pedigree information. This also confirmed by other studies, that a certain improvement can be achieved by adding a smaller reference population for traits with medium or high heritability [2, 43].

In this study, we investigated the performance of ML methods in genomic prediction, and demonstrated their superiority compared to classical methods GBLUP, ssGBLUP and Bayesian methods. Generally, the following characteristics of ML methods make it potentially attractive to genomic prediction. (I) Although ML methods generally require moderate fine-tuning of hyperparameters, the default hyperparameters usually do not perform poorly [34]. According to our results, ML methods after tuning parameters gained advantages over using the default hyperparameters; in addition, without tuning hyperparameters, almost all ML methods in CV and Adaboost.R2_KRR in predicting younger animals performed better than GBLUP and BayesHE (Tables 3, 4, 5). (II) ML methods can handle situations where the number of parameters is larger than the sample size, and they are very efficient in the case of high-density genetic markers for GS [44]. (III) ML methods do not make distribution assumptions about the genetic determinism underlying the trait, enabling us to capture the possible nonlinear relationships between genotype and phenotype in a flexible way [44], and it is different from GBLUP and Bayesian methods, which assume that all marker effects follow the same normal distribution or have different classes of shrinkage for different SNP effects. In addition, ML methods can take the correlation and interaction of markers into account as well, while linear models based on pedigree and genomic relationships may not provide a sufficient approximation of the genetic signals generated by complex genetic systems [16]. Consequently, for traits with fully additive architecture, conventional linear models outperformed ML models [45], but when traits are affected by nonadditive effects, especially epistasis, ML methods can achieve more accurate predictions [25]. These make ML methods gain a large advantage over GBLUP and BayesHE even though they only use genotyped animals.

In our experiments with 5-fold CV, our results showed that ML methods improved the prediction accuracy of the reproduction traits in the Chinese Yorkshire pig population. SVR, KRR, RF and Adaboost.R2 reflected the superiority of the ML methods, with average improvements over GBLUP of 20.5%, 21.0%, 14.1% and 20.5%, respectively. In predicting younger animals, our results also indicated that RF and Adaboost.R2_KRR gained 8.45% and 11.3% on TNB and 7.95% and 24.2% on NBA, respectively, over GBLUP. However, SVR and KRR did not perform as well as in CV, and ssGBLUP performed comparably with RF. Among ML methods, Adaboost.R2 performed consistently well in all situations; it generally outperformed ssGBLUP. Our findings related to ML methods were also confirmed in other studies. Liang et al. [46] also pointed out that compared with SVR, KRR, and RF, Adaboost possessed the most potent prediction ability in the genomic prediction of economic traits in Chinese Simmental beef cattle. Abdollahi-Arpanahi et al. [47] reported that the gradient boosting method yielded the best prediction performance in comparison with GBLUP, BayesB, RF, convolutional neural networks (CNN) and multilayer perceptron (MLP) in the genomic prediction of the sire conception rate (SCR) of Holstein bulls. Azodi et al. [48] compared the performance of six linear and five nonlinear ML models using data on 18 traits from six plant species and found that no one algorithm performed best across all traits, while ensemble learning performed consistently well.

In 5-fold CV, Adaboost.R2 and RF did not show the advantages of ensemble learning compared with single learning methods (SVR and KRR). For Adaboost.R2, mainly because the current SVR and KRR are sufficient to exert prediction abilities, which may limit the benefit of using boosting. In addition, the slow tuning process of Adaboost.R2, we did not precisely tune the hyperparameters, resulting in lower prediction accuracy than SVR and KRR. For RF, its prediction accuracy is mainly affected by the number and maximum depth of decision trees [46], but to weigh the practical application feasibility of RF, it is impractical to precisely tune the number of trees due to the slow tuning process. We obtained only approximate hyperparameters, leading to the most ideal RF model not being trained, further compromising its performance. In predicting younger animals, particularly for RF, they were precisely tuned based on the hyperparameter ranges of CV, resulting in the dramatic improvement of Adaboost and RF compared to SVR and KRR. Our results implied that ensemble learning is helpful to improve genomic prediction. Recently, another type of ensemble learning based on a hierarchical model also demonstrates advantages in genomic selection. Liang et al. [49] developed a stacking ensemble learning framework (SELF) that integrated SVR, KRR, and ENET to perform genomic prediction and showed excellent performance.

Our results indicated that tuning hyperparameters is necessary for ML methods, confirming that ML algorithms are sensitive to user-defined parameters during the training phase [37]. After tuning the hyperparameters in CV and in genomic prediction of younger individuals, the average improvement was 14.3% and 21.8% over those using default hyperparameters, respectively. The ML methods with optimal hyperparameters generally outperformed GBLUP and Bayesian methods, while they performed comparably with GBLUP and BayesHE in the case of default hyperparameters. On the other hand, our results also showed that the optimal hyperparameters depend on the characteristics of traits, datasets etc.. When optimal hyperparameters obtained in CV were used in predicting younger animals, the prediction accuracies of all ML methods were decreased compared to their performance with default parameters (Additional file 1: Table S1). In CV, many replicates were used for tuning hyperparameters, and the optimal hyperparameters were easily obtained for SVR and KRR due to their fast computing, while in predicting younger individuals, the hyperparameters were tuned based on only one genomic prediction, and they may not be sufficient to exert the generalization performance of SVR and KRR, leading to their relatively poorer prediction ability.

Moreover, our results indicated that the optimal hyperparameters may reduce the risk of overfitting (Tables 3, 4 and 5), which is a key element for the quality of the final predictions [50]. In this study, different ML models control overfitting with different parameters. For example, SVR mainly increases the fault tolerance of the model by increasing the regularization parameter C to achieve a regularization effect to reduce the degree of overfitting. KRR mainly tunes the hyperparameter λ that controls the amount of shrinkage to reduce noise, thereby controlling overfitting. For RF, the tendency of overfitting can be reduced by adding decision trees due to bagging and random feature selection, and the bias can be reduced by increasing the depth of the decision tree. Adaboost is an iterative algorithm, and each iteration weights the samples according to the results of the previous iteration; thus, with the continuation of iteration, the bias of the model will be continuously decreased. Accordingly, the tuning process highlights the flexibility of ML and increases the advantages of ML methods over conventional genomic selection methods.

Therefore, it is crucial to fine-tune the hyperparameters during the training phase when the dataset changes [16, 37, 48]. Meanwhile, it should be noted that the effect of the default hyperparameters usually did not perform poorly as discussed above, and failure to find suitable hyperparameters may greatly reduce the prediction effect of ML methods [46]. If hyperparameter automation can be realized during ML operation, it will greatly improve the efficiency of hyperparameter optimization and greatly broaden the application of ML methods in genomic prediction.

## Conclusions

In this study, we compared four ML methods, GBLUP, ssGBLUP and BayesHE to explore their efficiency in genomic prediction of reproduction traits in pigs. We compared the prediction accuracy, unbiasedness, MSE, MAE and computation time of different methods through 20 replicates of 5-fold CV and genomic prediction of younger animals. Our results showed that ML methods possess a significant potential to improve genomic prediction over that obtained with GBLUP and BayesHE. In 5-fold CV, ML methods outperformed conventional methods in all scenarios; they yielded higher accuracy and smaller MSE and MAE, while in genomic prediction of younger animals, RF and Adaboost.R2 performed better than GBLUP and BayesHE. ssGBLUP was comparable with RF and Adaboost.R2_KRR was overall better than ssGBLUP. Among ML methods, Adaboost.R2_KRR consistently performed well in our study. Our findings also demonstrated that tuning hyperparameters is necessary for ML methods, and the optimal hyperparameters depend on the characteristics of traits, datasets etc.

## Supplementary Information

**Additional file 1: Fig. S1.** Accuracy of genomic prediction obtained from the ssGBLUP method with different weighting factors, averaged by TNB and NBA and assessed by 20 replicates of 5-fold CV. **Table S1.** Accuracy and mean squared error (MSE) of genomic prediction of TNB and NBA from seven methods in predicting younger individuals using hyperparameters of CV.

## Data Availability

The datasets used or analyzed during the present study are available from the corresponding author on reasonable request.

## Abbreviations

GS: Genomic selection
GBLUP: Genomic BLUP
ssGBLUP: Single-step GBLUP
ML: Machine learning
RF: Random forest
SVR: Support vector regression
KRR: Kernel ridge regression
RKHS: Reproducing kernel Hilbert space
SVM: Support vector machine
TNB: Total number of piglets born
NBA: Number of piglets born alive
A matrix: Pedigree relationship matrix
EBV: Estimated breeding values
yc: Corrected phenotypes
DR2: Dosage R-squared measure
COR: Genotype correlation
CR: Genotype concordance rate
MAF: Minor allele frequency
BayesHE: Bayesian Horseshoe
CV: Cross validation
MSE: Mean squared error
MAE: Mean absolute error
KcRR: Cosine kernel-based KRR
SELF: Stacking ensemble learning framework
MCMC: Markov Chain Monte Carlo
