# Supplementary material for: Using machine learning to improve the accuracy of genomic prediction of reproduction traits in pigs
Source: J Anim Sci Biotechnol. 2022 May 17;13:60. doi: 10.1186/s40104-022-00708-0 (PMC9112588; doi:10.1186/s40104-022-00708-0)
Supplement: Supplementary file 1 — Additional file 1: Fig. S1. Accuracy of genomic prediction obtained from the ssGBLUP method with different weighting factors, averaged by TNB and NBA and assessed by 20 replicates of 5-fold CV. Table S1. Accuracy and mean squared error (MSE) of genomic prediction of TNB and NBA from seven methods in predicting younger individuals using hyperparameters of CV. [file 40104_2022_708_MOESM1_ESM.docx]

**
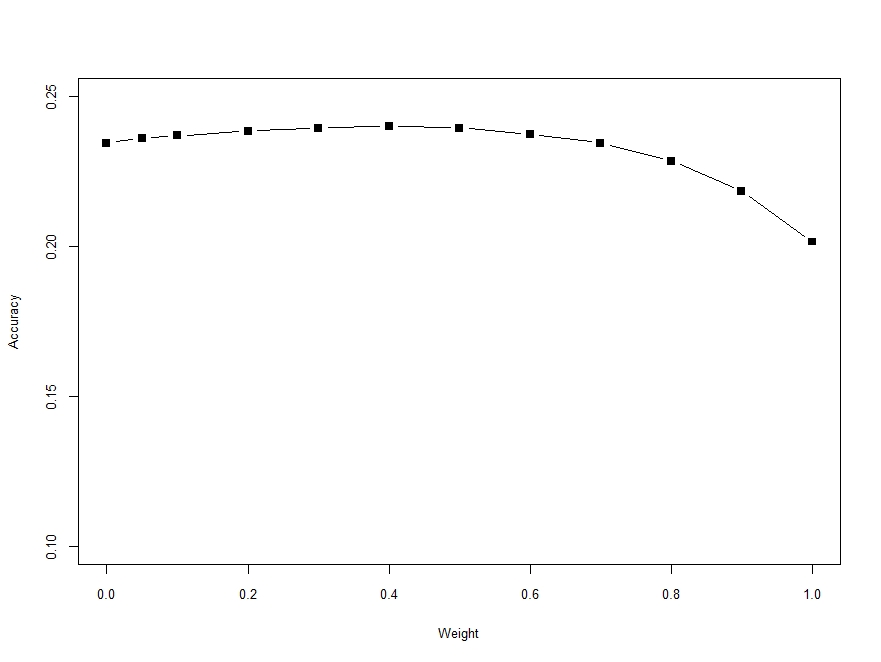
**

**Fig. S1** Accuracy of genomic prediction obtained from the ssGBLUP method with different weighting factors, averaged by TNB and NBA and assessed by 20 replicates of 5-fold CV.

**Table S1** Accuracy and mean squared error (MSE) of genomic prediction of TNB and NBA from seven methods in predicting younger individuals using hyperparameters of CV.

| Hyperparameters | Method | TNB^1^ | | NBA^2^ | |
| --- | --- | --- | --- | --- | --- |
|  |  | Accuracy^3^ | MSE | Accuracy^3^ | MSE |
|  | GBLUP | 0.355 | 11.598 | 0.264 | 10.203 |
|  | ssGBLUP | 0.408 | 11.221 | 0.288 | 9.974 |
|  | BayesHE | 0.357 | 11.566 | 0.262 | 10.143 |
| Optimal hyper-parameters of CV | SVR | 0.243 | 11.958 | 0.203 | 10.301 |
|  | KRR | 0.240 | 11.975 | 0.210 | 10.282 |
|  | RF | 0.270 | 11.894 | 0.260 | 10.201 |
|  | Adaboost.R2_KRR | 0.222 | 12.053 | 0.266 | 10.202 |
| Default | SVR | 0.271 | 11.858 | 0.17 | 10.370 |
|  | KRR | 0.346 | 11.538 | 0.259 | 10.116 |
|  | RF | 0.26 | 11.867 | 0.179 | 10.335 |
|  | Adaboost.R2_KRR | 0.36 | 11.392 | 0.322 | 9.794 |

^1^TNB: total number of piglets born

^2^NBA: number of piglets born alive

^3^Accuracy: the correlation between corrected phenotypes and predicted values of the validation population
